# Non-Coding Transcriptome Maps across Twenty Tissues of the Korean Black Chicken, Yeonsan Ogye

**DOI:** 10.3390/ijms19082359

**Published:** 2018-08-10

**Authors:** Hyosun Hong, Han-Ha Chai, Kyoungwoo Nam, Dajeong Lim, Kyung-Tai Lee, Yoon Jung Do, Chang-Yeon Cho, Jin-Wu Nam

**Affiliations:** 1 Department of Life Science, College of Natural Sciences, Hanyang University, Seoul 133791, Korea; yohae25@gmail.com (H.H.); nkw0228@gmail.com (K.N.); 2 Department of Animal Biotechnology & Environment of National Institute of Animal Science, RDA, Wanju 55365, Korea; hanha@korea.kr (H.-H.C.); lim.dj@korea.kr (D.L.); leekt@korea.kr (K.-T.L.); clonea@korea.kr (Y.J.D.); 3 College of Pharmacy, Chonnam National University, Kwangju 61186, Korea; 4 Animal Genetic Resource Research Center of National Institute of Animal Science, RDA, Namwon 55717, Korea; bloodtype@korea.kr; 5 Research Institute for Convergence of Basic Sciences, Hanyang University, Seoul 133791, Korea

**Keywords:** long non-coding RNA, chicken, black tissues, tissue-specific genes, non-coding transcriptome

## Abstract

Yeonsan Ogye is a rare Korean domestic chicken breed whose entire body, including feathers and skin, has a unique black coloring. Although some protein-coding genes related to this unique feature have been examined, non-coding elements have not been widely investigated. Thus, we evaluated coding and non-coding transcriptome expression and identified long non-coding RNAs functionally linked to protein-coding genes in Ogye. High-throughput RNA sequencing and DNA methylation sequencing were performed to profile the expression of 14,264 Ogye protein-coding and 6900 long non-coding RNA (lncRNA) genes and detect DNA methylation in 20 different tissues of an individual Ogye. Approximately 75% of Ogye lncRNAs and 45% of protein-coding genes showed tissue-specific expression. For some genes, tissue-specific expression levels were inversely correlated with DNA methylation levels in their promoters. Approximately 39% of tissue-specific lncRNAs displayed functional associations with proximal or distal protein-coding genes. Heat shock transcription factor 2-associated lncRNAs appeared to be functionally linked to protein-coding genes specifically expressed in black skin tissues, more syntenically conserved in mammals, and differentially expressed in black relative to in white tissues. Pending experimental validation, our findings increase the understanding of how the non-coding genome regulates unique phenotypes and can be used for future genomic breeding of chickens.

## 1. Introduction

The Yeonsan Ogye (Ogye) chicken is a breed of *Gallus gallus domesticus*. Domesticated in the Korean peninsula, this breed likely originated from the Indonesian Ayam Cemani black chicken, which populates tropical, high-temperature areas [1]. Ogye shares common features, such as black plumage, skin, shank, and fascia, with Ayam Cemani [1], although it has a smaller comb and shorter legs. Silkie fowl (Silkie), one of the most popular black-bone chickens, also has black skin but has white or varied color plumage [2]. Endothelin-3 (EDN3), which is involved in Silkie skin hyperpigmentation, has been reported in previous studies [2,3,4]. Recently, transcriptomes from Chinese native black chickens were compared to those from white chickens to globally identify hyperpigmentation-related genes [5]. Particularly, one hyperpigmentation-related gene, *EDN3*, was found to be duplicated in the fibromelanosis (FM) locus of both Silkie and Ayam Cemani [1,2]. However, studies of the molecular mechanisms and pathways related to black chicken hyperpigmentation have focused on coding genes.

A major part of the non-coding transcriptome corresponds to long non-coding RNAs (lncRNAs), which originate from intergenic, intervening, or antisense-overlapping regions of protein-coding genes [6,7,8]. LncRNAs are defined as transcripts longer than 200 nucleotides (nt) and are mostly untranslated because they lack an open reading frame; however, they interact with RNA-binding proteins and have diverse intrinsic RNA functions [9,10,11]. They tend to be localized in subcellular areas, particularly the nucleus, and often interact with heterochromatin remodelers and DNA methylation regulators to regulate gene expression at the epigenetic level.

LncRNAs are also known to regulate gene expression at the transcriptional, post-transcriptional, translational, and post-translational levels [9,10,12,13,14]. They regulate distant genes by modulating the recruitment of transcription factors (TFs) to target genes. However, few lncRNAs have been experimentally validated to be functional; most candidates remain unvalidated. Particularly, some lncRNAs were shown to regulate the expression of neighboring genes in a *cis*-acting manner [15,16,17,18,19]. Enhancer-associated lncRNAs (eRNAs) are a well-known group in this class that regulates the expression of downstream genes. Knockdown of eRNAs reduces target gene expression, suggesting that they function as *cis*-acting elements [20,21,22]. eRNA regulatory roles are achieved via several mechanisms: trapping TFs, directing chromatin roofing, and inducing DNA methylation [9,23,24,25,26,27]. In contrast, lncRNAs that associate with post-transcriptional regulators control target splicing and stability. For instance, antisense lncRNA from the FGFR2 locus promotes cell-type specific alternative splicing of *FGFR2* by interacting with the polycomb complex [28].

Despite their regulatory roles, few lncRNAs are highly conserved across vertebrates [29]. LncRNAs generally exhibit either poor conservation at the nucleotide level or conservation in a short region only, particularly compared to protein-coding genes [30,31,32]. Although sequence conservation often indicates related functions, it is difficult to detect conservation across multiple genome sequences because of technical challenges. LncRNAs, however, appear to be syntenically conserved with protein-coding genes, suggesting that lncRNAs have evolutionarily conserved roles in similar genomic contexts [33,34,35]. A zebrafish lncRNA, *linc-oip5*, which contains a short region of sequence conservation with mammalian orthologs in the last exon, also exhibits a preserved genomic architecture in its size and arrangement of exons; furthermore, *linc-oip5* loss of function disrupts zebrafish embryonic development, which can be rescued by mammalian orthologs [36]. Thus, examining the genomic context and/or short regions of conservation in lncRNAs may be necessary for understanding lncRNA function.

LncRNA expression signatures also provide insight into the functional roles of lncRNAs at the cellular level. Global lncRNA profiling demonstrated that lncRNAs generally exhibit lower expression than protein-coding genes [30,37,38] but tend to be uniquely or specifically expressed in distinct tissues, developmental stages, conditions, or disease states [29,30,31,37,39,40,41]. Additionally, large-scale analyses of lncRNA and protein-coding gene co-expression revealed that a considerable number of paired genes are co-regulated by common TFs [42,43]. Common TF binding motifs are frequently detected in the promoters of the co-expressed lncRNA and protein-coding genes, suggesting that the co-regulated genes share functional roles [44,45]. Thus, to predict lncRNA biological functions, co-expression networks of lncRNAs and protein-coding genes from large-scale transcriptomic data have been constructed and used for functional inference [46,47,48].

Although genome and transcriptome maps of livestock animals, such as rainbow trout, cow, goat, and chicken [49,50,51,52,53], were recently constructed, few non-coding transcriptome studies have been conducted in these genomes. To date, 9681 lncRNAs have been annotated in the red jungle fowl *Gallus gallus* genome according to the NONCODE database [54], but these studies were limited to a few tissues. Thus, in this study, high-throughput RNA sequencing and DNA methylation sequencing were performed in 20 different Ogye tissues. We profiled the expression landscape of protein-coding and lncRNA genes along with DNA methylation as well as identified lncRNAs functionally linked to protein-coding genes, some of which are specifically expressed in black skin tissues. Determining the expression landscape of Ogye non-coding transcriptomes in many tissues will improve the understanding of genomic similarities and differences between Ogye and other chickens.

## 2. Results

### 2.1. Tissue-Specific Expression and DNA Methylation Landscapes of Ogye lncRNAs

**Figure 1:**
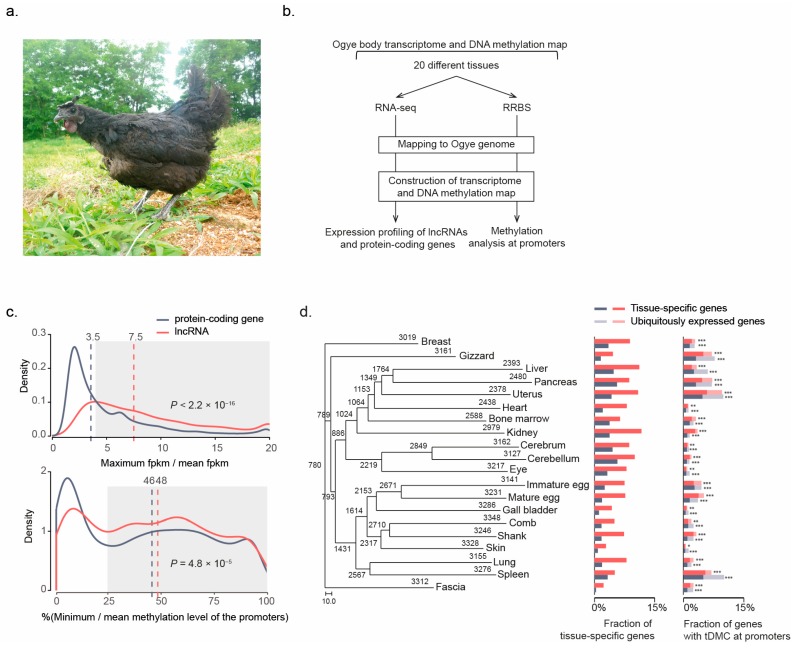
Expression and DNA methylation landscapes of Ogye lncRNAs. (**a**) Yeonsan Ogye. (**b**) A schematic flow for the analyses of coding and non-coding transcriptomes and DNA methylation from 20 different tissues. (**c**) Distributions of the maximum versus mean expression values of lncRNA (red line) and protein-coding genes (black line) across tissues (top), and distributions of the minimum versus mean methylation levels of each cytosine in the promoter of lncRNAs (red line) and protein-coding genes (black line) (bottom). The vertical dotted lines indicate the median value of the respective distribution (black for protein-coding genes and red for lncRNAs). The gray boxes indicate tissue-specific expression and methylation (**d**) Numbers of commonly or uniquely expressed lncRNAs across tissues are shown in the phylogenetic tree of tissues. The numbers at the leaf nodes indicate lncRNAs expressed in the indicated tissue (FPKM ≥ 1) and numbers at the internal nodes indicate those commonly expressed in the indicated tissues. Of the expressed genes in a certain tissue, the fraction of tissue-specific genes (red for lncRNA and black for protein-coding genes) and fraction of genes with a differentially methylated region (DMR) in the promoters are indicated as bar graphs. Of the genes with a DMR, tissue-specific genes (dark) and others (light) were distinguished and the enrichment of tissue-specific genes was tested using Fisher’s exact test (* *p* ≤ 1 × 10^−5^, ** *p* ≤ 1 × 10^−10^, *** *p* ≤ 1 × 10^−20^). The scale bar represents 10.0, which is the unit of 120 differentially expressed genes across tissues.

Although our recent study showed that the fibromelanosis (FM) locus containing the hyperpigmentation-related *EDN3* gene is duplicated in the Ogye genome [55], to identify functional lncRNAs related to black chicken hyperpigmentation, we profiled the expression of lncRNA genes by RNA-seq across 20 Ogye tissues (Figure 1b; see “Expression profiling” section in the Methods and Supplementary methods for more details;). Of the 6900 Ogye lncRNAs that we annotated in our previous study [55], 6565 were expressed with fragments per kilobase of transcript per million mapped reads (FPKM) ≥ 1 in at least one tissue, whereas 13,765 of Ensembl chicken protein-coding genes (release 81; http://www.ensembl.org/biomart) were expressed. Tissue-specific genes with a four-fold higher maximum expression value than the mean value over the 20 tissues were depicted in the genome using a Circos plot (Supplementary Figure S1a, green track). As previously reported [31,56,57], Ogye lncRNAs generally display tissue-specific expression pattern with a four-fold higher maximum expression value than the mean value over the 20 tissues. Some lncRNAs were expressed in only a single tissue, although 25% of lncRNA genes (1709 loci) displayed ubiquitous expression across tissues. Approximately 75% of lncRNA genes (5191 loci) were tissue-specific, a significantly higher proportion than that of protein-coding genes (45%; Figure 1c; top; Supplementary Table S1; *p* < 2.2 × 10^−16^; Fisher’s exact test). The fractions of tissue-specific lncRNAs ranged from 2.4% (fascia) to 12.5% (kidney), which are much higher than the percentages recorded for protein-coding genes, which ranged from 0.4% (fascia) to 4.2% (kidney) (Figure 1d; middle). Hierarchical clustering of commonly expressed lncRNA genes among tissues using the PHYLIP package (version 3.6) [58] (see “Hierarchical clustering of expressed lncRNAs across tissues” in the Methods for more details) defined functionally and histologically-related tissue clusters. Particularly, 2317 lncRNAs were specifically expressed in the comb, skin, and shank, which are black tissues in Ogye (Figure 1d; left). Only 780 lncRNAs were ubiquitously expressed in all tissues (Figure 1d; left).

To correlate tissue-specific lncRNA expression with its epigenetic status in the respective tissue, DNA methylation signals were profiled from corresponding tissues by reduced representation bisulfite sequencing (RRBS; see the “Datasets” section in the Methods and Supplementary methods for more details). A significant correlation (nominal *p* ≤ 0.05; false discovery rate (FDR) = 0.18) between the expression levels and methylation signals in the region 2 kb upstream (regarded as the promoter) of genes across the 20 tissues was demonstrated, along with variations in methylation (Supplementary Figure S1a), with a ratio of the minimum to mean methylation levels at CpG sites in a corresponding promoter. Approximately 70% of lncRNA genes containing CpG methylation signals in their promoter region displayed tissue-specific methylation with a 25% lower minimum methylation value than the mean value over the 20 tissues, which is a significantly higher proportion than that of protein-coding genes (64%; Figure 1b; bottom; *p* = 4.8 × 10^−5^; Fisher’s exact test). Taken together, lncRNAs are more tissue-specific, both in expression and DNA methylation, than protein-coding genes.

To examine the association between gene expression and promoter methylation, the genes with tissue-specific differentially methylated CpG sites (see the “Tissue-specific, differentially methylated CpG sites” section in the Methods for more details) that include ≥5 reads with C to T changes in ≥10 tissues in the promoter region were subjected to downstream analyses. The fractions of genes showing tissue-specific differentially methylated CpG sites in their promoters were significantly enriched in tissue-specific genes (Figure 1d; right). Of the lncRNA and protein-coding genes with tissue-specific differentially methylated CpG sites, 6.4% of lncRNAs and 9.3% of protein-coding genes displayed a significant negative correlation (nominal *p* ≤ 0.05) between their promoter methylation levels and expression levels; these percentages were significantly higher than those of random-pair controls (Supplementary Figure S1b; *p* = 1.30 × 10^−6^ for lncRNAs; *p* = 7.93 × 10^−36^ for protein-coding genes; Fisher’s exact test). However, only approximately 3% of genes showed a positive correlation between their expression and methylation signals, which is comparable to or less than in the control (Supplementary Figure S1c; *p* = 0.87 for lncRNAs; *p* = 0.013 for protein-coding genes). Collectively, these results suggest that CpG methylation in the promoters is associated with the expression of target genes.

### 2.2. Tissue-Specific lncRNA Clusters Functionally Linked to Protein-Coding Genes

**Figure 2:**
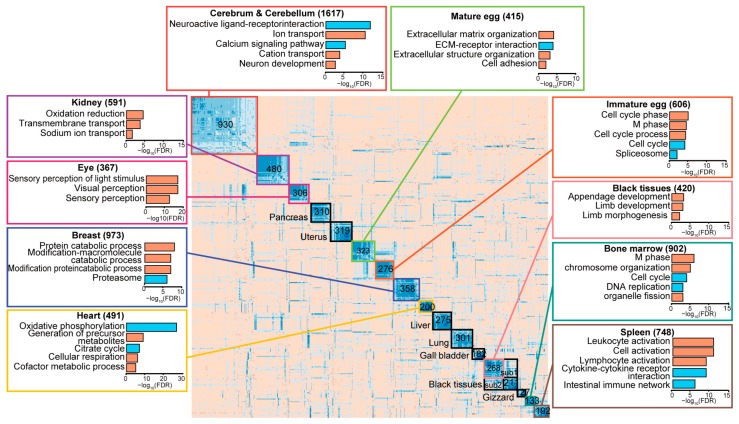
Co-expression clusters of lncRNAs across 20 tissues based on Pearson’s correlation coefficient metrics using ‘rsgcc’ package in R and functional annotations (see the “Clustering of co-expressed lncRNAs” section in the Methods for more details). Co-expression clustering of lncRNAs across 20 tissues defines 16 clusters and two sub-clusters specific to a tissue or a set of similar tissues. The boxes outlined in a color indicate those with significant gene ontology (GO) biological processes (orange bars) or Kyoto Encyclopedia of Genes and Genomes (KEGG) pathway terms (cyan bars) associated with protein-coding genes co-expressed with lncRNAs in the respective cluster. The significant enrichment of terms was tested using the hypergeometric test and adjusted by the FDR, indicated using a logarithmic scale on the *X*-axis in the box. Clusters outlined in black are those with neither a significant association with a GO term nor any co-expressed protein-coding genes. Sub-clusters in the clusters are indicated where appropriate. The number in each cluster indicates the number of lncRNAs in the cluster and the number in the boxes with functional terms indicates the number of co-expressed protein-coding genes.

As lncRNAs tend to be specifically expressed in a tissue or in related tissues, they may provide functional evidence for defining the phenotypic characteristics of tissues. To identify functional clusters of lncRNAs, pairwise correlation coefficients between tissue-specific lncRNAs were calculated and the co-expression patterns across the 20 tissues were clustered, defining 16 co-expression clusters (Figure 2). As expected, each co-expression cluster was defined as a functional group, highly expressed in a specific tissue (kidney, eye, pancreas, uterus, mature egg, immature egg, breast, heart, liver, lung, gall bladder, gizzard, bone marrow, or spleen) or related tissues (brain and black tissues) (Supplementary Table S2). Particularly, the largest co-expression cluster, the brain-specific group, contained 930 co-expressed lncRNAs highly expressed in the cerebrum and cerebellum. The second largest cluster, the black tissue-specific group, contained 479 co-expressed lncRNAs highly expressed in the fascia, comb, skin, and shank (Figure 2). Clusters of related tissues also displayed distinct sub-modules corresponding to each tissue. For instance, lncRNA clusters specific to black tissues displayed sub-clusters including sub-cluster 1 specific to the shank and sub-cluster 2 specific to the comb, although the sub-clusters shared skin-specific expression (Supplementary Figure S2).

The functional role of each co-expressed lncRNA cluster can be indirectly inferred from a set of co-expressed mRNAs [46,47,48]. Thus, mRNAs that are exclusively co-expressed with each lncRNA cluster were identified based on the following criteria: an average Pearson’s correlation coefficient ($\bar{r}$) ≥ 0.5 with members within a cluster and differences between the corresponding $\bar{r}$ and the mean correlation (${\bar{r}}_{i}$) with all other groups ≥0.3, which were subsequently subjected to gene ontology (GO) analyses using DAVID [59] (Figure 2; Supplementary Table S3). Particularly, 1617 mRNAs exclusively correlated with the brain-specific lncRNA group (930 lncRNAs) were identified and associated with brain-function specific terms, such as neuroactive ligand-receptor interaction (*q* = 2.18 × 10^−12^; FDR correction). In contrast, 748 mRNAs exclusively correlated with spleen-specific lncRNAs were identified and associated with immune-related terms, such as leukocyte activation (*q* = 2.37 × 10^−12^). Similarly, 10 of 16 co-expression clusters of lncRNAs showed GO enrichment, with significantly enriched GO terms and KEGG pathways (Figure 2).

### 2.3. LncRNAs as Epigenetic Activators

**Figure 3:**
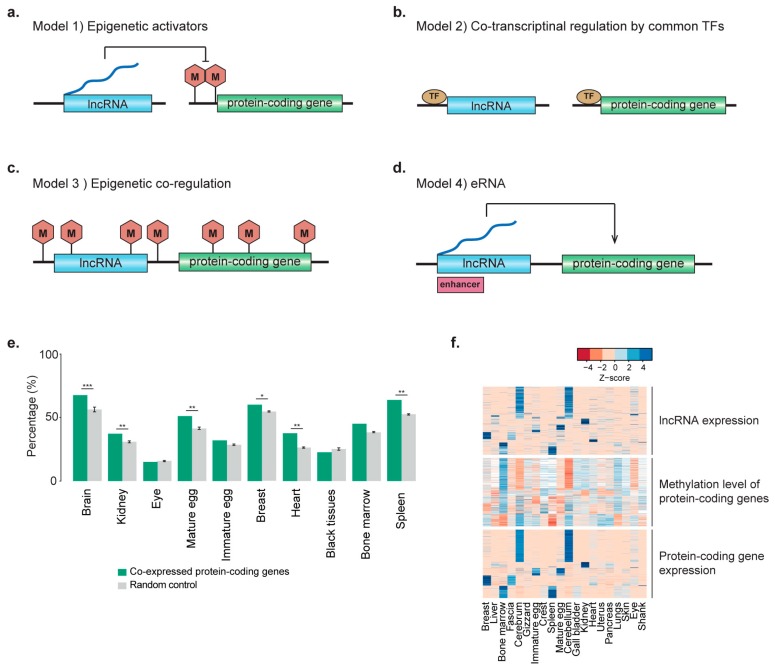
Models of lncRNA and protein-coding gene co-regulation and lncRNAs as epigenetic activators. (**a**) LncRNAs as epigenetic activators that suppress the methylation level in the promoter of protein-coding genes. (**b**) Transcriptional co-regulation of lncRNA and protein-coding genes by common TFs. (**c**) Epigenetic co-regulation of neighboring lncRNA and protein-coding genes. (**d**) eRNAs that activate the expression of neighboring protein-coding genes. (**e**) Proportions of lncRNAs with expression levels correlated with the methylation level in the promoter of co-expressed protein-coding genes (dark green) in each cluster are shown in bar graphs. The numbers were compared to the mean methylation level of randomly selected protein-coding genes. To test the significance of the enrichment of lncRNAs as epigenetic activator candidates, 1000 number-matched random cohorts were compared to the original numbers (* *p* ≤ 0.05, ** *p* ≤ 0.01, *** *p* ≤ 0.001). (**f**) LncRNAs as epigenetic activators whose expression levels are negatively correlated with the methylation level in the promoters of protein-coding genes, which in turn are negatively correlated with the level of protein-coding gene expression, as shown in the heatmaps. The key indicates the z-score range of the expression values. White indicates N.A.

The coherent expression of two different RNA classes may be important in the outcome of either active regulation by lncRNAs in *cis* and *trans* or co-regulation by common regulators, such as TFs or epigenetic regulators in *cis* and *trans* (Figure 3a–d). Regulation of gene expression by lncRNAs often involves engagement with chromatin remodelers, such as polycomb repressive complexes that mediate the suppression of target mRNA expression [60,61] or demethylases that open the chromatin structure to enhance the expression of target mRNAs [62,63] (Figure 3a). Remote co-expression of lncRNAs and mRNAs can be also regulated by common TFs [42,43] (Figure 3b). Co-expressed genes tend to have common TF binding motifs in their promoters. However, *cis*-regulation of mRNA expression by lncRNAs is known to be associated with common epigenetic factors (Figure 3c) or enhancers (Figure 3d).

To identify potential lncRNAs that act as epigenetic activators to reduce methylation levels, lncRNAs with expression levels significantly negatively correlated with the methylation level in the promoters of co-expressed protein-coding genes (nominal *p* ≤ 0.01) were examined in each co-expression cluster. In this case, the lncRNAs were thought to reduce the methylation level in the promoters of co-expressed protein-coding genes. Of the lncRNAs in clusters, expression of 15.0–72.9% indicated a significantly negative correlation with methylation levels in the promoters of co-expressed protein-coding genes, which were compared to those of random protein-coding gene cohorts (Figure 3e). Clusters specific to the brain, kidney, mature egg, breast, heart, and spleen included significantly more lncRNAs with a significant correlation compared to random controls (*p* = 0.026–7.71 × 10^−13^), but this was not observed for the black tissue cluster. To further identify potential lncRNAs associated with the DNA methylation status of target genes, we also examined whether the expression and methylation of co-expressed coding genes were correlated (nominal *p* ≤ 0.01). A total of 820 lncRNAs in the clusters were identified as confident DNA methylation activator candidates (Figure 3f). Genes encoding for lncRNAs that act as DNA methylation regulators of protein-coding genes were mostly 100 kb apart, and only five were within 100 kb of the target genes, suggesting that lncRNAs that function as epigenetic activators mostly function in *trans*-form rather than in *cis*-form.

### 2.4. Transcriptional Regulation by Common TFs

**Figure 4:**
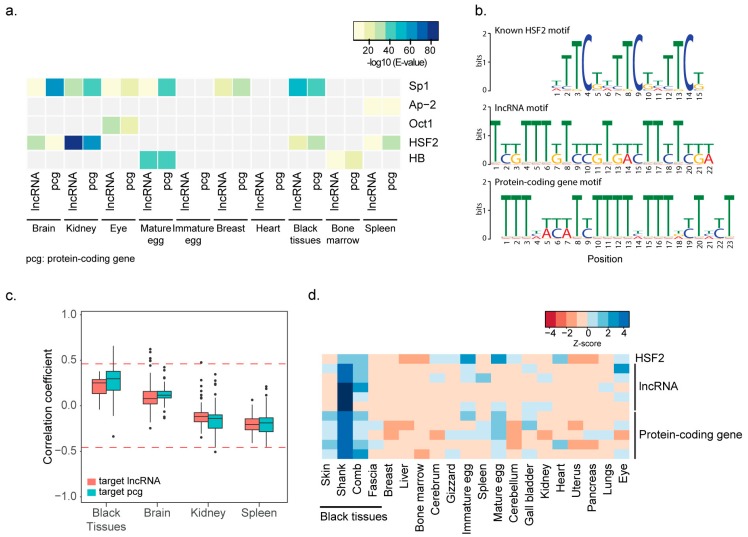
Co-transcriptional regulation of lncRNA and protein-coding genes by common TFs. (**a**) TFs (Sp1, Ap-2, Oct1, HSF2, and HB) with binding motifs significantly co-enriched in the promoters of lncRNAs in a tissue-specific cluster and their co-expressed protein-coding genes are shown in the heatmap. The TFs are expressed in the indicated tissues. The significance of motif enrichment was tested using MEME and E values are presented with color codes (blue: more significant, yellow: less significant) in the key. PCG indicates protein-coding gene. (**b**) HSF2 binding motif. A known motif is shown in the top panel, motif in lncRNA promoters is shown in the middle panel, and motif in protein-coding gene promoters is shown in the bottom panel. (**c**) The expression correlation between co-regulated genes (red boxes for lncRNAs and green boxes for protein-coding genes) and HSF2 across tissues. Red lines indicate the significance level of the correlation coefficient (*p* ≤ 0.05). (**d**) Expression pattern of *HSF2* and its target genes showing the top 5 correlations with *HSF2*.

To identify co-expressed pairs of lncRNAs and mRNAs regulated by common TFs, TF binding sites (TFBSs) enriched in the promoters of co-expressed genes were examined. Because the TFBS annotations of chicken are incomplete and TFBS databases were largely derived from mammalian sequences, we used a de novo method to detect potential TFBS motifs. For this analysis, sequences 2 kb upstream of the co-expressed genes were extracted and enriched sequence motifs were identified using the multiple expectation-maximization for motif elicitation (MEME) suite [64] (see the “Prediction of TFBSs” section in the Methods for more details). The resulting motifs were subjected to analysis by the TOMTOM program [65] to annotate TFBSs based on the TRANSFAC database v3.2 [66]. As a result, 14 common TFs with significantly abundant binding sites in the promoters of lncRNA and protein-coding genes were detected (Supplementary Figure S3; corresponding to model 2). To discern TFs available in chicken genomes, PANTHER [67,68] was used to examine detect the presence of chicken orthologs of the TFs and determine whether the orthologs are expressed in the corresponding tissues (FPKM ≥ 1). Finally, five orthologous, expressed TFs including HSF2 and SP1, were identified as candidates (Figure 4a). HSF2 and SP1 binding sites were more recurrently detected across tissues than others and significantly enriched in the promoters of 478 lncRNAs and 634 protein-coding genes. Although the binding motifs were slightly degenerated from the annotated motifs, the HSF2 motifs were similar in the promoters of lncRNA genes and protein-coding genes (Figure 4b).

To examine further whether the respective TFs affect the expression of lncRNAs and protein-coding genes, the correlation between the expression of each TF and co-expressed genes in each cluster was examined. Interestingly, *HSF2* expression showed a strong positive correlation with gene expression in black tissues but not in other tissues (Figure 4c). The expression pattern for each of the five lncRNAs and protein-coding genes that were highly correlated with that of *HSF2* was specific for the skin, shank, and comb compared to other tissues (Figure 4d). Thus, HSF2 may be a candidate for regulating the black tissue-specific expression of lncRNAs and protein-coding genes. Taken together, our data indicate that of a total of 3466 lncRNA in 10 clusters, 615 (17.74%) appeared to be co-regulated with co-expressed protein-coding genes by common TFs, such as HSF2.

### 2.5. Coherent Expression of Neighboring lncRNA and Protein-Coding Genes

**Figure 5:**
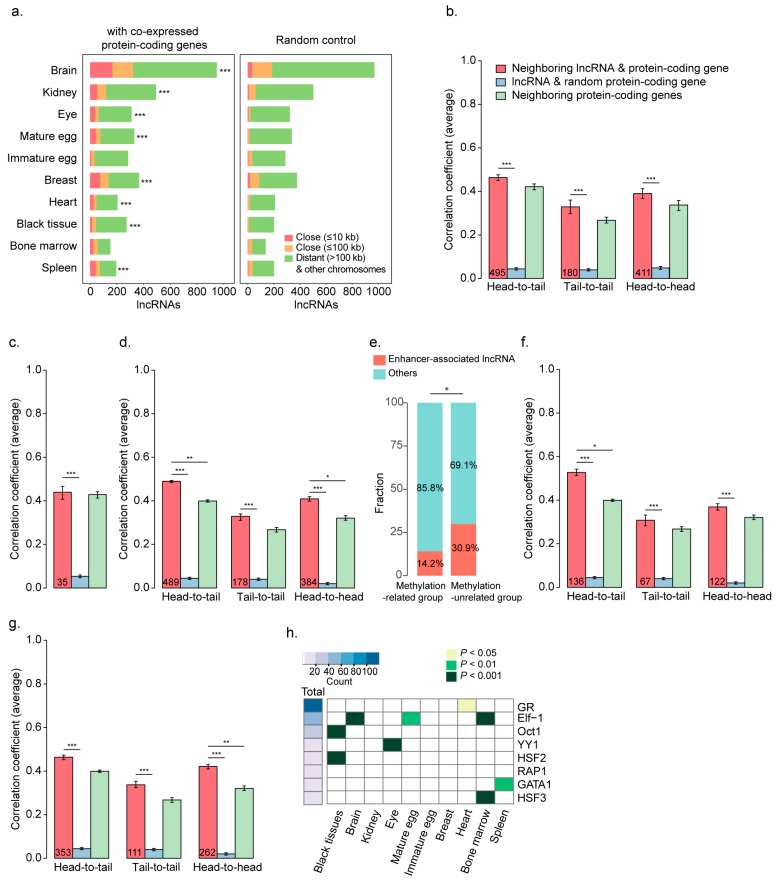
Co-regulation of neighboring lncRNA and protein-coding genes. (**a**) Numbers of lncRNAs, classified by distance from the closest protein-coding gene (red for the ≤10 kb group, orange for the ≤100 kb group, and green for the >100 kb or on another chromosome group) (left). *, **, and *** indicate *p* ≤ 0.05, ≤0.01, and ≤0.001, respectively. (**b**) The average correlation coefficients of tissue-specific lncRNA and protein-coding gene pairs in close neighborhoods (≤10 kb) are shown based on their relative orientations (head-to-tail, tail-to-tail, and head-to-head) (red bars). The average correlation coefficients of random pairs are also shown (blue bars) and those of tissue-specific protein-coding gene pairs in close neighborhoods (≤10 kb) are shown with green bars. *, **, and *** indicate *p* ≤ 0.05, ≤0.01, and ≤0.001, respectively. Error bars indicate the standard error. The number in the bars indicates the number of analyzed pairs. (**c**) The average correlation coefficients of neighboring lncRNA and protein-coding genes with similar methylation levels in their promoters (methylation-related) are shown in bar graphs. Otherwise, as in (**b**). The bar colors correspond to (**b**). (**d**) The average correlation coefficients of tissue-specific lncRNA and protein-coding genes (methylation-unrelated), except for those of (**c**). Otherwise, as in (**b**). Bar colors correspond to (**b**). (**e**) Proportion of eRNAs (red) in the methylation-related group (**c**) and methylation-unrelated group (**d**). ** indicates *p* ≤ 0.01. (**f**) Average correlation coefficients of tissue-specific eRNAs. Otherwise, as in (**b**). Bar colors correspond to (**b**). (**g**) Average correlation coefficients of tissue-specific lncRNAs not associated with enhancers. Otherwise, as in (**b**). Bar colors correspond to (**b**). (**h**) TF binding motifs significantly associated with the eRNAs. The total count of the indicated TF binding sites in eRNAs is indicated in the heatmap (left) and the significance of the association over the total background is indicated with color-coded *p* values across tissues. The significance of a specific TF binding motif was tested using a binomial test in each tissue.

Previous studies showed that lncRNAs and their neighboring protein-coding genes are highly correlated in their expression across tissues and developmental stages [34,37]. To examine how the co-expressed lncRNAs and mRNAs in our study are co-localized in chromosomes, lncRNAs from each group were first classified based on the closest distances (≤10, ≤100, >100 kb, and other chromosomes) from significantly co-expressed protein-coding genes (nominal *p* ≤ 0.01; Pearson’s correlation) (Figure 5a). Genes encoding co-expressed pairs of lncRNAs and mRNAs were significantly proximally co-localized within 10 kb (Figure 5a left; *p* ≤ 0.05, Fisher’s exact test) compared to in random controls (Figure 5a right) but not those of lncRNAs and mRNAs in the range of 10–100 kb or greater than 100 kb. Overall, 2–15% of the co-expressed pairs in the clusters tended to be proximally co-regulated within 10 kb.

To examine how neighboring lncRNAs and protein-coding genes are tissue-specifically co-regulated, pairs within 10 kb were classified into three categories based on their relative orientations (head-to-tail, tail-to-tail, or head-to-head). The correlation coefficients of the pairs in each category were compared to those of lncRNA and random protein-coding gene controls from tissue-specific gene sets (Figure 5b) or from ubiquitously expressed gene sets (Supplementary Figure S4a). Both neighboring lncRNA and protein-coding gene pairs displayed significantly greater correlations than did random controls, regardless of the category, in both sets (Figure 5b; Supplementary Figure S4a). The correlations were also compared to those of neighboring protein-coding gene pairs. While the correlations of ubiquitously expressed, neighboring lncRNAs and protein-coding genes were significantly lower than those of ubiquitously expressed neighboring protein-coding gene pairs in the head-to-tail and head-to-head categories (Supplementary Figure S4a), the correlation coefficients of tissue-specific pairs were slightly but insignificantly higher than those of neighboring protein-coding gene pairs (Figure 5b).

To dissect the factors affecting co-regulation of tissue-specific neighboring lncRNA and protein-coding gene pairs, pairs with a high correlation (*p* ≤ 0.05) between the methylation levels of their promoters (methylation-related group, model 3) and those with no correlation (methylation, unrelated group) were divided. Tissue-specific neighboring lncRNA and protein-coding gene pairs showed no greater expression correlation than did neighboring protein-coding genes in the methylation-related group (Figure 5c; *p* = 0.71, Wilcoxon rank sum test), whereas they showed a significantly higher correlation in the methylation-unrelated group (Figure 5d; *p* ≤ 0.001 for head-to-tail, *p* ≤ 0.05 for head-to-head, Wilcoxon rank sum test), suggesting that neighboring lncRNAs and protein-coding genes in the methylation-unrelated group exhibit a regulatory interaction.

### 2.6. Enhancer-Associated RNA-Mediated Gene Regulation

Previous studies showed that lncRNAs associated with enhancers can regulate their neighboring protein-coding genes [69]. The genomic associations between lncRNAs and enhancers, detected in embryonic developmental stages in the chicken [70], revealed that lncRNAs in the methylation-unrelated group are more significantly associated with enhancers than those in the other group (Figure 5e; *p* = 2.72 × 10^−6^; Fisher’s exact test). As a result, 136 head-to-tail lncRNAs, 67 tail-to-tail lncRNAs, and 124 head-to-head lncRNAs were considered as enhancer-associated lncRNA candidates (eRNAs; Supplementary Table S4). The eRNAs (corresponding to model 4) showed a greater correlation with neighboring protein-coding genes only in the head-to-tail group (Figure 5f), whereas non-eRNAs displayed a greater correlation in the head-to-head orientation, which may allow sharing of common promoters (Figure 5g). A few eRNAs were found to have strong bi-directional transcriptional activity (Supplementary Figure S4b; see the “Transcriptional activity of eRNAs” section in the Methods for more details), as previously reported [20,71]

Next, to identify TFs binding to genomic regions that transcribe eRNAs, TFBSs detected from all genomic regions associated with enhancers were profiled and compared to those of TFs detected from enhancers specific to a certain tissue (Figure 5h). Oct1 and HSF2 binding sites were significantly localized in eRNAs specific to black tissues (*p* < 3.09 × 10^−5^ for Oct1; *p* < 3.11 × 10^−7^ for HSF2; binomial test). In addition to TFs specific to black tissues, GR, YY1, RAP1 and GATA1, and HSF3 binding sites were localized in eRNAs specific to the heart, eye, spleen, and bone marrow, respectively (Figure 5h). Interestingly, HSF2 was a common TF candidate for co-regulating lncRNAs and protein-coding genes from a distance (Figure 5d).

### 2.7. Conserved Black Skin-Specific lncRNAs

**Figure 6:**
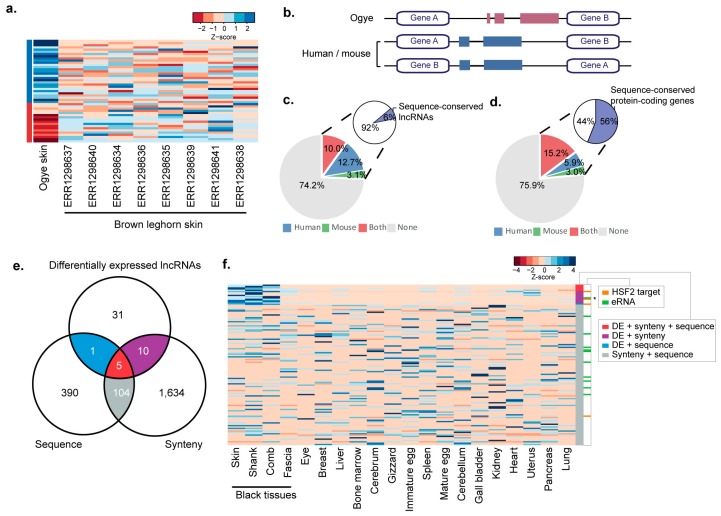
Black tissue-specific lncRNAs with sequence and synteny conservation. (**a**) Expression patterns of differentially expressed lncRNAs in Ogye skin compared to Brown leghorn skin samples. Expression levels are indicated with a color-coded Z-score (red for low and blue for high expression) as shown in the key. (**b**) Cartoon showing a lncRNA that is syntenically conserved with upstream and downstream protein-coding genes in the human and/or mouse genome. (**c**) The fraction of lncRNAs with syntenic conservation in the human (blue), mouse (green) or both (red) genomes is shown in the pie chart. Of the syntenically conserved lncRNAs, the fraction of lncRNAs with sequence conservation (purple) in the human or mouse genome is indicated in the secondary pie charts. (**d**) The fraction of protein-coding genes with synteny conservation is indicated in the pie chart. Otherwise, as in (**c**). (**e**) The numbers of differentially expressed lncRNAs in black skin with evidence of sequence and synteny conservation are indicated in a Venn diagram. Functional evidence for differential expression (DE) + synteny + sequence (red), DE + synteny conservation (purple), or DE + sequence conservation (blue) are indicated in the Venn diagram. (**f**) Sixteen blackskin-specific lncRNAs are shown in a heatmap with functional evidence (colors correspond to (**e**)) A total of 104 non-specific lncRNAs with evidence of sequence + synteny conservation are indicated in gray. The co-regulation models associated with a certain lncRNA are indicated to the left with color codes (orange for HSF2 binding and green for eRNAs). * indicates the eRNA associated with HSF2. The expression level is indicated with a color-coded z-score, as shown in the key.

As described above, unlike other chicken breeds, both the plumage and skin of the Ogye are black. To identify lncRNAs potentially functionally related to this trait, lncRNAs specifically co-expressed in black tissues (Figure 2) were further investigated by comparison with those in non-black skin of other chicken breeds. Of the 479 lncRNAs specific to black tissues, 47 were significantly up-regulated (29) or down-regulated (18) by at least two-fold in Ogye black skin compared to in Brown leghorn skin (Figure 6a; Supplementary Table S5; FDR < 0.05).

To identify functionally conserved lncRNAs, the 47 differentially expressed lncRNAs were examined for synteny and sequence conservation in the human and mouse genomes. Synteny conservation considers whether orthologs of a certain lncRNA’s neighboring genes are positionally conserved in these mammalian genomes (Figure 6b). The results showed that approximately 10% of lncRNAs were syntenically conserved in both the human and mouse genomes and approximately 25% were syntenically conserved in at least one genome (Figure 6c; Supplementary Table S6), percentages that are comparable to those of protein-coding genes (Figure 6d). However, sequence similarity analyses by BLAST showed that only 6% of syntenically conserved lncRNAs had conserved sequences relative to sequences in either the human or mouse genomes (Figure 6c; Supplementary Table S6), which is much lower than that of protein-coding genes (56%). Taken together, our data show that 16 lncRNAs were syntenically or sequentially conserved and differentially expressed in black tissue (Figure 6e).

**Figure 7:**
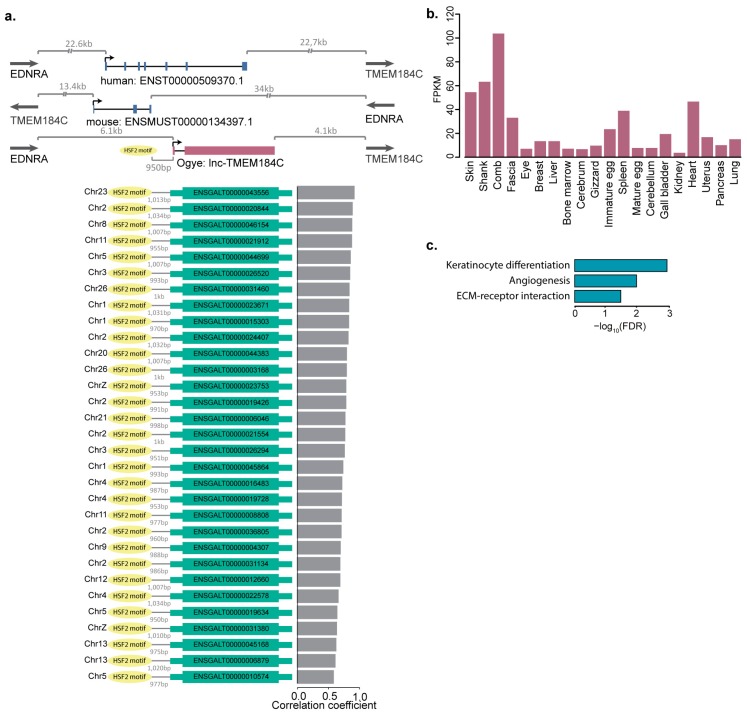
Example of black skin-specific lncRNAs with synteny conservation, which is transcriptionally regulated by HSF2. (**a**) Ogye lncRNA (*lnc-TMEM184C*) with synteny conservation in human and mouse genomes (top). The lncRNA has an HSF2 binding motif in its promoter; this motif is also present in the promoters of protein-coding genes with correlated expression (below). Gray bar plots indicate the expression correlation between the lncRNA and protein-coding genes. (**b**) *Lnc-TMEM184C* expression pattern across 20 tissues. (**c**) GO terms significantly associated with the protein-coding genes co-expressed with *lnc-TMEM184C*.

Of the 16 lncRNAs showing evidence of black tissue-specific function, four, including eRNAs, were associated with HSF2-binding motifs, whereas of the 104 showing synteny and sequence conservation but not differential expression in black tissues, only one was associated with *HSF2*. The presence of HSF2-binding motifs appears to be significantly related to black tissue-specific expression (Figure 6f; *p* ≤ 0.0008, Fisher’s exact test). For instance, *linc-THEM184c* was significantly up-regulated in black tissue (Figure 7b) and its locus is syntenically conserved with neighboring genes; *TMEM184C* and *EDNRA*, in both the human and mouse genomes, and its promoter contains an HSF2-binding motif (Figure 7a). Additionally, the protein-coding genes co-expressed with this lncRNA were enriched for GO terms that are functionally relevant to black skin: keratinocyte differentiation, angiogenesis, and ECM-receptor-interaction (Figure 7c). Among the co-expressed genes, 31 have HSF2-binding sites in their promoters (Figure 7a). As another example, black tissue-specific linc-*FAM204A* is syntenically conserved with the *RAB11FIP2* and *FAM204A* genes in the human and mouse genomes (Supplementary Figure S5a). This lncRNA was highly expressed in black tissues including the skin, shank, and comb but showed no expression in other tissues except for the eye (Supplementary Figure S5b). The co-expressed protein-coding genes were enriched for the functionally relevant GO terms melanogenesis, ECM-receptor interaction, and Wnt signaling (Supplementary Figure S5c). Interestingly, the human and Ogye lncRNA orthologs share a conserved sequence of 389 nt, which includes multiple miRNA 7-mer target sites (Supplementary Figure S5a).

## 3. Discussion

In this study, we determined the specific expression patterns of lncRNAs that co-regulated with protein-coding genes across 20 tissues of Ogye and possible regulatory mechanisms for the co-regulation. However, it is difficult to generalize these findings because our analyses were performed using one individual. Therefore, our findings should be either experimentally validated or further examined at the population level.

Most lncRNAs showed a tissue-specific expression pattern, defining functionally coherent co-expression clusters. The tissue-specific expression and coherent expression of lncRNA genes with other protein-coding genes may be attributed to common epigenetic and transcriptional regulation. In fact, of the lncRNAs in clusters, 39.3% were predicted to be associated with at least one model (Supplementary Figure S6a); most commonly, these involved lncRNAs that act as epigenetic activators of protein-coding gene expression and common TFs that bind to the lncRNA and protein-coding gene promoters (Supplementary Figure S6b). Interestingly, 126 lncRNAs had evidence supporting both the epigenetic activator and TF models (Supplementary Figure S6c). Seventy-nine lncRNAs had functional evidence supporting their identities as eRNAs. Although lncRNAs are known to be mostly involved in epigenetic repression of genes, our study focused on lncRNAs as epigenetic activators by correlating the level of lncRNAs and methylation in target gene promoters. Furthermore, because only a subset of CpG sites may be related to the chromatin state and transcriptional activity of target genes, averaging CpG methylation signals in the promoter may have underestimated the fraction of epigenetically activating lncRNAs in our study.

Although protein-coding genes co-expressed with lncRNAs in black tissues appear to be not associated with epigenetic regulation by DNA methylation (Figure 3a), lncRNA and protein-coding genes co-expressed in black tissues are enriched with HSF2-binding sites in their promoters and were specifically correlated with HSF2 levels across tissues, supporting that the genes are co-regulated by HSF2 (Figure 4). Moreover, enhancers that included HSF2-binding sites were associated with eRNAs specific to black tissue (Figure 5h). One black skin-specific lncRNA, *lnc-THMEM184c*, was most abundantly expressed in the comb, and HSF2 appears to co-regulate *lnc-THMEM184c* and its co-expressed protein-coding genes, which are related to keratinocyte differentiation and ECM-receptor interactions (Figure 7).

In addition, several previous studies focusing on animal coat color showed that color can be determined by the amount and type of melanin produced and released by melanocytes present in the skin [72,73]. Melanin is produced by melanosomes, large organelles in melanocytes, in a process known as melanogenesis. Wnt signaling plays a regulatory role in the melanogenesis pathway and is required for the developmental process that leads to melanocyte differentiation from neural crest cells [74,75]. One candidate lncRNA related to this process is *linc-FAM204A*, whose co-expressed protein-coding genes are associated with the GO terms melanogenesis, ECM-receptor interaction, and Wnt signaling pathway (Supplementary Figure S5c). *Linc-FAM204A*, which contains a short-conserved motif, is broadly preserved in mammalian genomes, including the human, rhesus macaque, mouse, dog, and elephant genomes. Among these orthologs, the human ortholog is known as *CASC2*, and is suppressed in lung, colorectal, renal, and other cancers by *miR-21-5p* targeting via the conserved 7-mer site (Supplementary Figure S5a).

Taken together, these results indicate that coding and non-coding RNAs functionally relevant to black and other tissues can help explain the unique genomic and functional characteristics of a Korean domestic chicken breed, Yeonsan Ogye. Additionally, these findings provide insight for future studies of industrial and agricultural applications, such as the use of genetic markers in genomic breeding for selecting economically beneficial breeds, as well as for comparative analysis of chicken genomes.

## 4. Materials and Methods

### 4.1. Acquisition and Care of Yeonsan Ogye

Yeonsan Ogye chickens (object number: 02127), obtained from the Animal Genetic Resource Research Center of the National Institute of Animal Science (Namwon, Korea), were used in this study. The care and experimental use of Ogye was reviewed and approved by the Institutional Animal Care and Use Committee of the National Institute of Animal Science (IACUC No.: 2014-080; approval date: 4 July 2014). Ogye management, treatment, sample collection, and further analysis of all raw data were performed at the National Institute of Animal Science.

### 4.2. Datasets

To profile the expression of protein-coding genes in the Ogye genome, *Gallus gallus* (red junglefowl) protein-coding genes were downloaded from Ensembl biomart (release 81; http://www.ensembl.org/biomart) and mapped onto the Ogye draft genome v1.0 using GMAP (v2015-07-23) [76]. Genes showing greater than 90% coverage and identity were selected as Ogye protein-coding genes. As a result, 14,264 protein-coding genes were subjected to further analysis.

Total RNA samples and bisulfite-treated DNA samples were collected from 20 different tissues (breast, liver, bone marrow, fascia, cerebrum, gizzard, immature egg, comb, spleen, mature egg, cerebellum, gall bladder, kidney, heart, uterus, pancreas, lung, skin, eye, and shank) from 8-month-old Ogye (Figure 1a). Approximately 1.5 billion RNA-seq reads (843 million single-end reads and 638 million paired-end reads) and 123 million RRBS reads were analyzed (Figure 1a).

### 4.3. Tissue-Specific, Differentially Methylated CpG Sites

RRBS reads were aligned to the Ogye draft genome (v1.0) [55] using Bismark [77]. The methylation level of each cytosine in a CpG region was calculated using Bismark methylation extractor. A tissue-specific, differentially methylated CpG site is defined as one in which its mean methylation across tissues is at least four-fold greater than the minimum signal in a specific tissue. The tissue-specific, differentially methylated CpG sites were found in the promoter region of each gene, defined as the region 2 kb upstream of the 5′ end of genes.

### 4.4. Expression Profiling

The expression values (FPKM) of lncRNA and protein-coding genes were estimated using RSEM (v1.2.25) in each tissue. The values across tissues were normalized using the quantile normalization method. In all downstream analyses, lncRNA or protein-coding genes with FPKM ≥ 1 in at least one tissue were used. LncRNAs for which the maximum expression value across the 20 tissues was at least four-fold higher than the mean value were considered to exhibit tissue-specific expression.

### 4.5. Hierarchical Clustering of Expressed lncRNAs across Tissues

To perform hierarchical clustering of commonly expressed lncRNA genes among tissues, the list of expressed lncRNAs in each tissue was used as an input vector for hierarchical clustering. Clustering was conducted using the PHYLIP package. LncRNAs with FPKM ≥ 1 in a specific tissue were considered to be expressed in that tissue. As two tissues share more common genes, they become more closely clustered.

### 4.6. Clustering of Co-Expressed lncRNAs

Hierarchical clustering was performed to search for expression clusters of lncRNAs across the 20 tissues using Pearson’s correlation coefficient metrics. Clusters in which more than 80% of their members were most highly expressed in the same or related tissues (brain and black tissues) were regarded as tissue-specific. Sub-clusters in the brain and black tissue clusters were further defined using the criterion described above.

### 4.7. Defining Coding Genes Co-Expressed with lncRNAs in a Cluster

Protein-coding genes with a high mean correlation with lncRNAs in a cluster (Pearson’s correlation ≥ 0.5), but for which the mean correlation to the cluster was at least 0.3 greater than those of other clusters, were assigned to the co-expressed set of the cluster. Each set of mRNAs was used to perform GO term and pathway enrichment analyses using DAVID [59]. Terms were only selected when the FDR *q* value was ≤0.05.

The correlation of the methylation level of neighboring lncRNA and protein-coding genes was analyzed.

The methylation levels at CpG sites in the promoters of neighboring lncRNA and protein-coding genes were correlated with each other over the 20 tissues (based on Pearson’s correlation coefficients). Only tissues in which a specific position had sufficient read coverage (at least five) were considered for measuring the correlation. If the nominal *p* value was ≤0.05, the pair of lncRNA and protein-coding genes was considered to have a significantly correlated interaction.

### 4.8. Correlating the Expression Level of lncRNAs with the Methylation Level of Protein-Coding Genes

To identify lncRNAs as potential epigenetic activators, the expression of lncRNAs and the methylation at CpG sites in the promoters of protein-coding genes were correlated over twenty tissues using a non-parametric correlation method (Spearman’s correlation). Only pairs of lncRNA and protein-coding genes exhibiting a nominal *p* value ≤ 0.01 were considered as having a significantly correlated interaction. Of the resulting pairs, if the protein-coding mRNAs had a significant correlation (nominal *p* value ≤ 0.01) between their expression level and the methylation level in their promoter, its paired lncRNA was regarded as an epigenetic activator.

### 4.9. Prediction of TFBSs

To identify enriched TFBSs in the promoters of co-expressed lncRNAs in each tissue-specific cluster and promoters of co-expressed protein-coding genes within the cluster, the promoter sequences were examined using the MEME suite (V4.9.0; http://meme-suite.org/). Motifs showing an *E*-value ≤ 1 × 10^−5^ were selected as enriched motifs, which were associated with the corresponding tissue. The resulting motifs were searched in the TRANSFAC database [66] using TomTom [65].

### 4.10. Identification of Enhancer Regions

To annotate enhancer regions in the Ogye draft genome, annotation files including all enhancers in the *Gallus gallus* (red junglefowl) genome were downloaded from the NCBI gene expression omnibus (GEO; https://www.ncbi.nlm.nih.gov/geo/; GSE75480). Enhancer sequences extracted using our in-house script were aligned to the Ogye draft genome using blastn. Regions that significantly matched the original enhancers (*E*-value ≤ 1 × 10^−5^) and with high coverage of greater than 80% were annotated as Ogye enhancers.

### 4.11. Transcriptional Activity of eRNAs

To examine the bi-directional transcriptional activity of eRNAs, total mapped reads in the range spanning 1 kb upstream to 1 kb downstream of the eRNA transcription start site were re-examined on both the forward and reverse strands.

### 4.12. Correlation of Expression between Neighboring lncRNA and Protein-Coding Genes

Pairs consisting of an lncRNA and its closest neighboring protein-coding gene within 10 kb were classified into three groups based on their genomic orientations: head-to-head (can be divergently overlapped), head-to-tail (including only independent lncRNAs with evidence of a transcription start site and cleavage and polyadenylation site; otherwise, these lncRNAs must be at least 1 kb apart from each other), and tail-to-tail (can be convergently overlapped). The correlation of the expression of these pairs was calculated over the 20 tissues using Pearson’s correlation method. The average correlation coefficient values and their standard errors were calculated in the respective groups. As a random control, the expression of 1000 random pairs of lncRNA and protein-coding genes were correlated using the same method. As another control, number-matched pairs of neighboring protein-coding genes were correlated with each other.

### 4.13. Synteny and Sequence Conservation

To examine the conservation of synteny of an lncRNA, its closest downstream and upstream neighboring protein-coding genes in the Ogye genome were matched to their orthologous genes in the mouse and human genomes. If an lncRNA was located between the two orthologous genes, regardless of direction, that lncRNA was regarded as syntenically conserved. GENCODE lncRNA annotations (v25 for human and vM11 for mouse) were analyzed in this study. To detect sequence conservation, Ogye lncRNA sequences were aligned to lncRNA sequences from other species, intronic sequences, and their flanking sequences (up to 4 Mb) using BLAST. For a significant match, an *E*-value 1 × 10^−6^ was used as a cutoff.

### 4.14. Analysis of lncRNA Differential Expression

To identify lncRNAs differentially expressed between Ogye and Brown leghorn skin tissues, Brown leghorn skin RNA-seq data were downloaded from the NCBI SRA (https://www.ncbi.nlm.nih.gov/sra; ERR1298635, ERR1298636, ERR1298637, ERR1298638, ERR1298639, ERR1298640, and ERR1298641). Reads were mapped to the *Gallus gallus* Galgal4 reference genome using Bowtie (v1.0.0) [78], and the average mismatch rates were estimated across read positions. If the mismatch rate was greater than 0.1 at a certain position, sequences on high mismatch side of the position were trimmed using seqtk (https://github.com/lh3/seqtk), and then sickle was used with the default option for read quality control. Preprocessed reads from RNA-seq data were mapped onto the chicken Galgal4 reference genome using STAR (v2.4.2) [79].The read counts of lncRNAs were performed using HTSeq (v0.6.0) [80] and the average read count of lncRNAs from Brown leghorn skin tissues was compared to that of Ogye (one replicate). Differential expression analysis was performed using the negative binomial test of DESeq [81]. Genes showing a greater than two-fold difference in expression and FDR adjusted *p* value ≤ 0.05 were considered as differentially expressed.

### 4.15. Data Availability

Raw RNA-seq and RRBS data from the 20 different Ogye tissues have been submitted to the NCBI GEO (https://www.ncbi.nlm.nih.gov/geo/; GSE104351) under SuperSeries accession number GSE104358. All lncRNA catalogs and expression tables from this study have also been submitted to NCBI GEO (GSE104351) under the same SuperSeries accession number, GSE104358.

## Supplementary Materials

Supplementary materials can be found at http://www.mdpi.com/1422-0067/19/8/2359/s1.
