# Supplementary material for: Non-Coding Transcriptome Maps across Twenty Tissues of the Korean Black Chicken, Yeonsan Ogye
Source: Int J Mol Sci. 2018 Aug 10;19(8):2359. doi: 10.3390/ijms19082359 (PMC6121550; doi:10.3390/ijms19082359)
Supplement: Supplementary file 1 [file ijms-19-02359-s001.zip › Supplementary files/Supplementary figures.pdf]

Non-coding transcriptome maps across twenty tissues of Korean black chicken, Yeonsan Ogye

Hyosun Hong[1,6], Han-Ha Chai[3,4,6], Kyoungwoo Nam[1], Dajeong Lim[3], Kyung-Tai Lee[3], Yoon Jung Do[3], Chang-Yeon Cho[5] and Jin-Wu Nam[1,2,*]

[1]Department of Life Science, College of Natural Sciences, Hanyang University, Seoul 133791, Republic of Korea
[2]Research Institute for Convergence of Basic Sciences, Hanyang University, Seoul 133791, Republic of Korea
[3]Department of Animal Biotechnology & Environment of National Institute of Animal Science, RDA, Wanju 55365, Republic of Korea
[4]College of Pharmacy, Chonnam National University, Kwangju 61186, Republic of Korea
[5]Animal Genetic Resource Research Center of National Institute of Animal Science, RDA, Namwon 55717, Republic of Korea
[6]These authors contributed equally to this paper.

Supplementary Figure 1

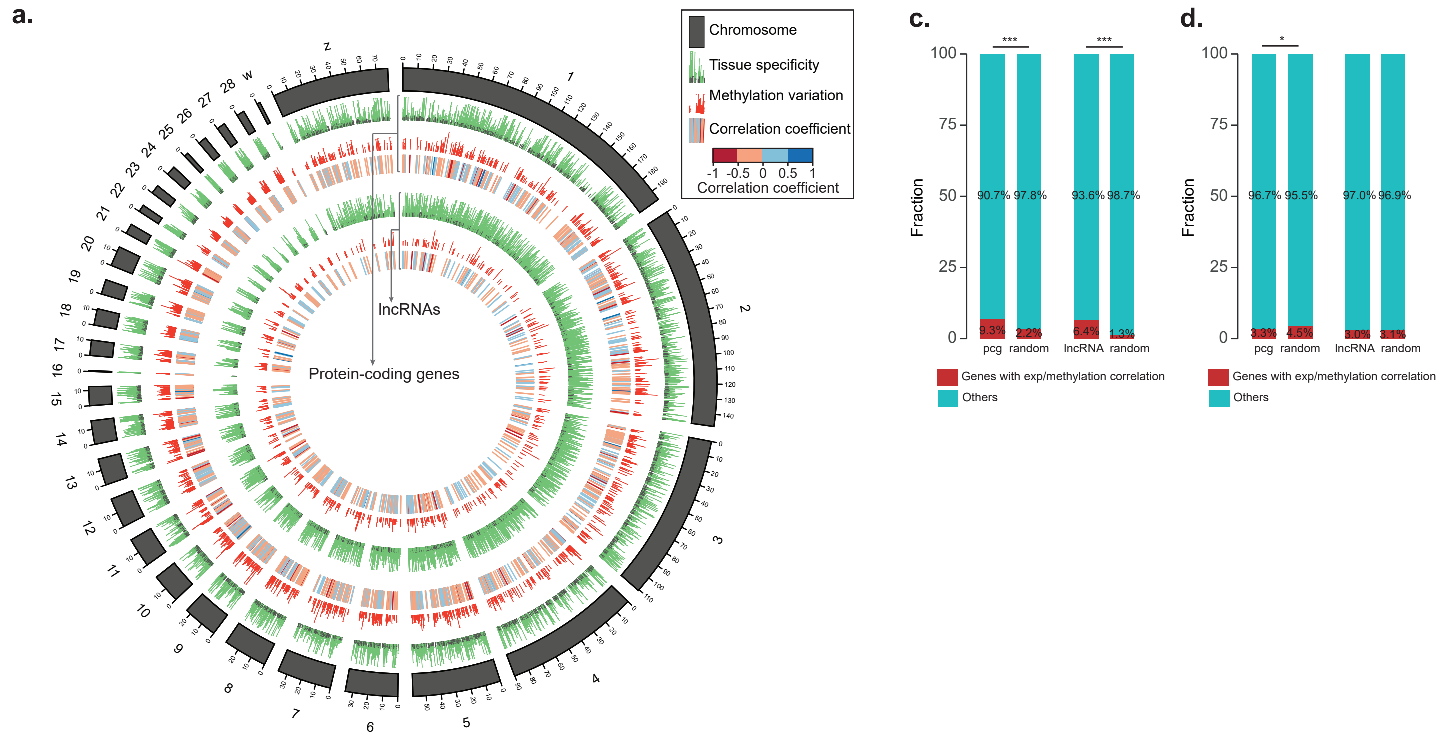

Supplementary Figure 1. a. A Circos plot illustrating the expression variability (green bars) of lncRNA and protein-coding genes, the methylation variability (red bars) at tissue-specific, differentially methylated CpG sites in the promoters, and the correlation coefficients between expression and methylation levels across chromosomes (heatmaps). The variability was measured as the ratio of the maximum and mean expression values or methylation levels. The correlation was calculated with Spearman correlation coefficients, which were demonstrated with a heatmap, scaled from -1 to 1. b. The proportion of genes (protein-coding genes (left) and lncRNAs (right)) with a significant negative correlation (red) between the methylation level in their promoters and their expression values is shown. the enrichment was tested using Fisher's exact test (* P ≤ 0.05, ** P ≤ 0.01, *** P ≤ 0.001). c. The proportion of genes with a significant positive correlation between the methylation level and their expression values is shown. Otherwise, as in b.

Supplementary Figure 2

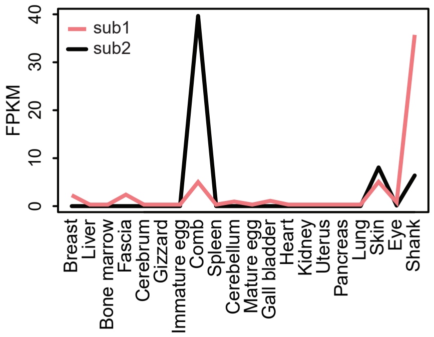

Supplementary Figure 2. Expression patterns of sub-clusters (sub 1 for shank, 2 for comb) in the black tissue cluster.

Supplementary Figure 3

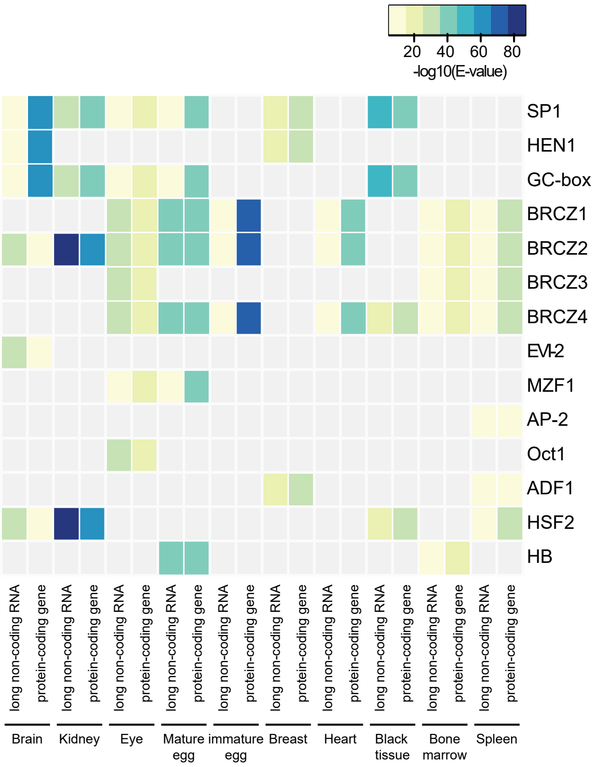

Supplementary Figure 3. 14 TF binding motifs significantly co-enriched in the promoters of lncRNAs in a tissue-specific cluster and their co-expressed protein-coding genes are shown in the heatmap (E-value ≤ $1X10^{-5}$).

Supplementary Figure 4

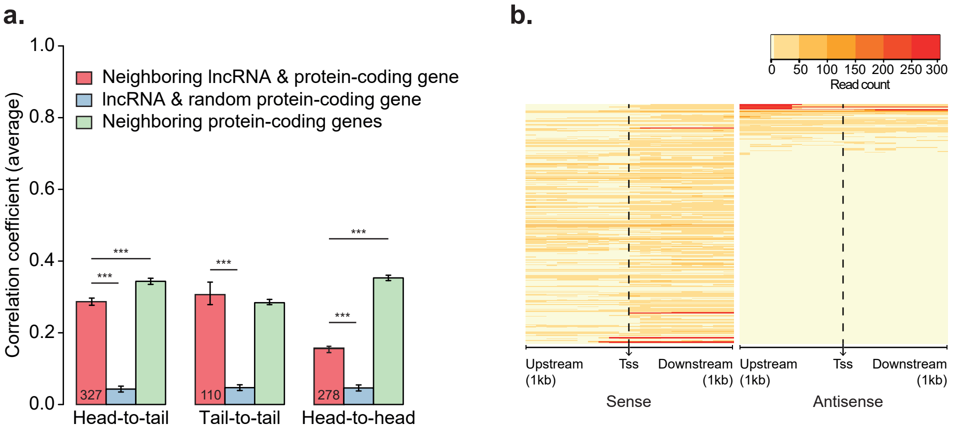

Supplementary Figure 4. a. Average correlation coefficients of ubiquitously expressed lncRNAs and neighboring protein-coding genes. Otherwise, as in Fig. 6b. b. The read counts are indicated with color codes (described in the key) in the sense (left) and antisense (right) strands based on the relative position from the eRNA TSS. Yellow indicate no read.

Supplementary Figure 5

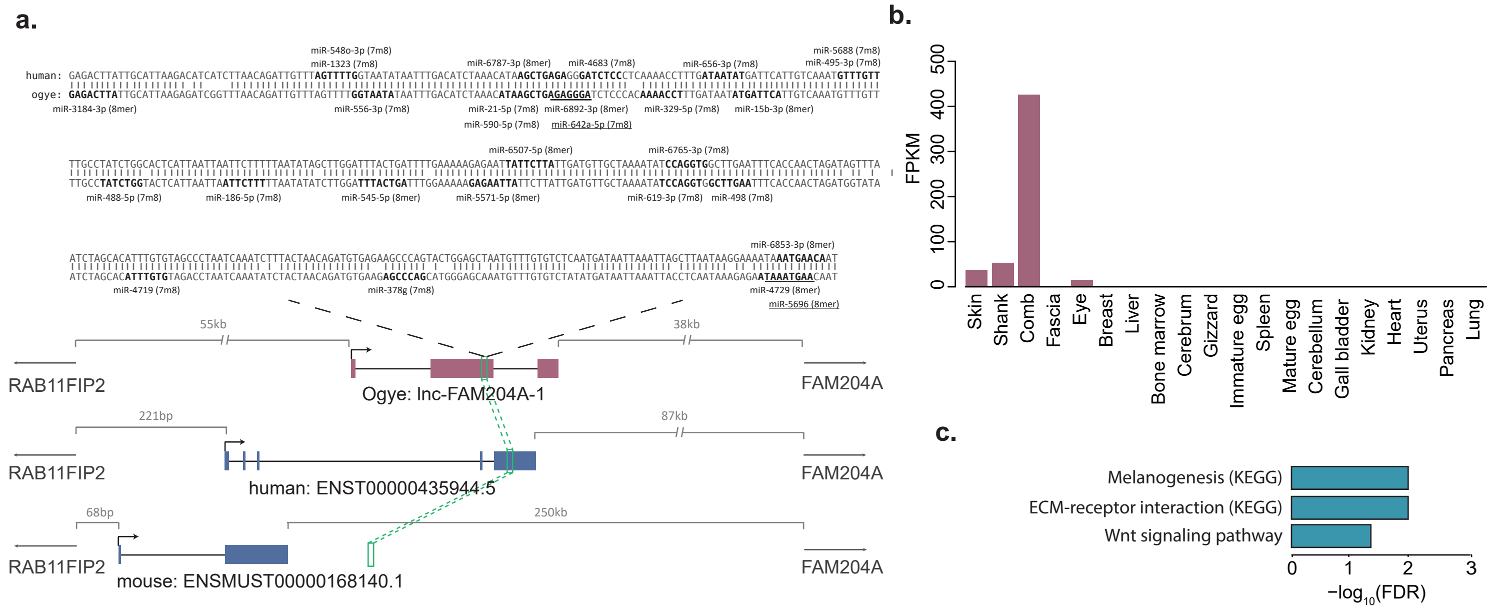

Supplementary Figure 5. a. An example of an Ogye lncRNA (linc-FAM204A) that contains a sequence that is conserved in the exonic region (green box) of human lncRNA ENST00000435944.5 but not in the corresponding mouse gene. It is also syntenically conserved with sequences in both the mouse and human genome. 7-mer mRNA target sites in the conserved region are indicated in the sequences (top). b. Expression pattern of linc-FAM204A. c. GO terms that are significantly associated with protein-coding genes that are co-expressed with linc-FAM204A.

Supplementary Figure 6

a.

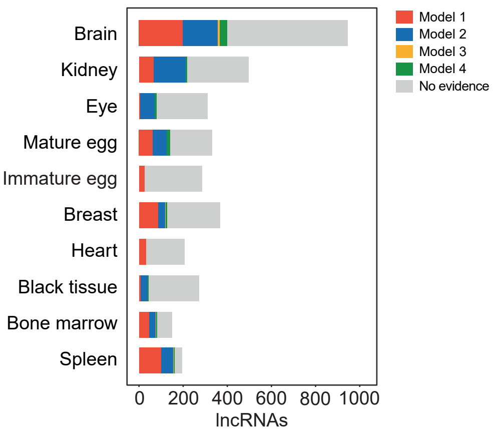

b.

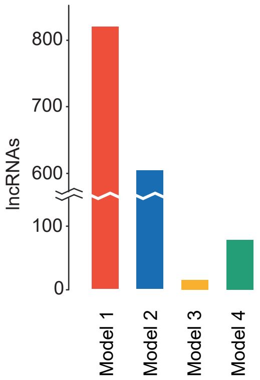

c.

Model	Count
model1 & model2 & model4	5
model1 & model2	126
model1 & model3	5
model1 & model4	15
model2 & model3	3
model2 & model4	8

Supplementary Figure 6. a. The numbers of lncRNAs, associated with specific clusters, with characteristics that are explained by the different co-regulation models are indicated in the stacked bar graphs (red for epigenetic activator (model1); blue for co-transcriptional regulation by TFs (model 2); yellow for epigenetic co-regulation (model 3); green for eRNA-mediated regulation (model 4); grey for no associated model. b. The numbers of lncRNAs from all clusters with characteristics that are explained by each model. c. The numbers of lncRNAs with characteristics that are supported by more than two model.
