# Supplementary material for: Non-Coding Transcriptome Maps across Twenty Tissues of the Korean Black Chicken, Yeonsan Ogye
Source: Int J Mol Sci. 2018 Aug 10;19(8):2359. doi: 10.3390/ijms19082359 (PMC6121550; doi:10.3390/ijms19082359)
Supplement: Supplementary file 1 [file ijms-19-02359-s001.zip › Supplementary files/supplementary method.docx]

**Supplementary Methods**

**Preparation of RNA-seq libraries:** Total RNAs were extracted from twenty Ogye tissues using 80% EtOH and TRIzol. The concentration of the isolated RNA was measured using Quant-IT RiboGreen (Invitrogen, Carlsbad, USA). The RNA integrity was detected using a TapeStation RNA screentape (Agilent, Waldbronn, Germany). An RNA Integrity Number (RIN) value of the samples that was greater than seven could be used in sequencing. Each library was independently prepared with 300ng of total RNA using an Illumina TruSeq Stranded Total RNA Sample Prep Kit (Illumina, San Diego, CA, USA). The rRNA was removed using a Ribo-Zero kit from total RNA. After rRNA depletion, the remaining RNA was purified, fragmented and primed for cDNA synthesis. The cleaved RNA fragments were copied into the first cDNA strand using reverse transcriptase and random hexamers. This step was followed by second strand cDNA synthesis using DNA Polymerase I, RNase H and dUTP. The resulting cDNA fragments then underwent an end repair process, the addition of a single ‘A’ base, after which adapters were ligated. The products were purified and enriched with PCR to create the final cDNA library. The libraries were quantified using qPCR according to the qPCR Quantification Protocol Guide (KAPA Library Quantificatoin kits for Illumina Sequecing platforms) and qualified using the TapeStation D1000 ScreenTape assay (Agilent Technologies, Waldbronn, Germany).

**Preparation of RRBS libraries:** Preparation of reduced representation bisulfite sequencing (RRBS) libraries was done following Illumina’s RRBS protocol. 5ug of genomic DNA was digested with the restriction enzyme MspI and purified with a QIAquick PCR purification kit (QIAGEN, Hilden, Germany). Digested DNA was subjected to library preparation using TruSeq Nano DNA Library Prep Kit (Illumina, San Diego, USA). Eluted DNA fragments were end-repaired, extended on the 3′ end with an ‘A’, and ligated with Truseq adapters. After ligation had been assessed, the products, which ranged from 175 to 225bp in length (insert DNA of 55–105 bp plus adaptors of 120 bp), were excised from a 2%(w/v) Low Range Ultra Agarose gel (Biorad, Hercules, USA) and purified using the QIAquick gel extraction protocol. Then bisulfite conversion was performed using an EpiTect Bisulfite Kit (Qiagen, 59104), and the bisulfite-converted DNA libraries were amplified by PCR (four cycles) using PfuTurbo Cx DNA polymerase (Agilent, 600410). The final product was then quantified using qPCR and qualified using the Agilent Technologies 2200 TapeStation assay (Agilent, Waldbronn, Germany). The final product was sequenced using the HiSeq™ 2500 platform (Illumina, San Diego, USA).
